# Higher‐Order Interactions Can Promote Coexistence by Rewiring Intransitivities Into Competitive Networks

**DOI:** 10.1111/ele.70415

**Published:** 2026-06-30

**Authors:** Zachary Hajian‐Forooshani, Ivette Perfecto, Warren Irizarry, John Vandermeer

**Affiliations:** ^1^ German Centre for Integrative Biodiversity Research (iDiv) Halle‐Jena‐Leipzig Leipzig Germany; ^2^ Department of Computer Science Martin Luther University Halle‐Wittenberg Halle (Saale) Germany; ^3^ School for Environment and Sustainability University of Michigan Ann Arbor Michigan USA; ^4^ Department of Ecology and Evolutionary Biology University of Michigan Ann Arbor Michigan USA

**Keywords:** ants, coexistence, competition, higher‐order interactions, host‐parasitoid interaction, intransitivity, phorids, trait‐mediated indirect interaction

## Abstract

Higher‐order interactions (HOIs) are widely predicted to promote coexistence, yet the underlying ecological mechanisms behind this effect remain largely unexplored in natural communities. Here, we integrate natural history and theory to show how HOIs can restructure competitive networks and influence coexistence. With over 2 years of data on a tropical ant community, we estimate pairwise competitive interactions and demonstrate that, in isolation, they fail to recreate observed community dynamics. We find that inclusion of an HOI, imposed by a parasitoid of the dominant species, can theoretically reorganize competitive networks to mirror the dynamics of the empirical system. We demonstrate how temporal variation in HOIs forces the community between dominance regimes and that the interregnum between regimes is riddled with competitive intransitivities that promote coexistence. This work provides an empirical example of the ecological mechanisms behind the coexistence‐promoting effects of HOIs and suggests that HOIs and intransitivity, which are typically treated separately, can be mechanistically linked through the rewiring of species interactions.

## Graphical abstract

We integrate natural history and theory to show how higher‐order interactions (HOIs) can restructure competitive networks and influence coexistence in a tropical ant community. The HOI from a parasitoid of the dominant ant species forces the community to move between two dominance regimes, and the interregnum between regimes has multiple interacting competitive intransitivities. We provide an empirical example of the ecological mechanisms behind the coexistence‐promoting effects of HOIs and suggest that HOIs and intransitivity can be mechanistically linked through the rewiring of species interactions.

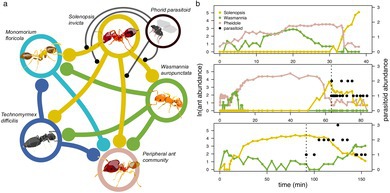

## 1 Introduction

Competition has arguably been the focal abstraction for ecologists attempting to understand the underlying mechanisms of species coexistence. Gause's formalization of the competitive exclusion principle (Gause 1934) helped place competition at the center of explanations for community organization at both ecological (MacArthur 1958; Hutchinson 1959) and evolutionary timescales (MacArthur and Levins 1967). Much of this important classical work had two, often implicit, yet fundamental assumptions—first, that community structure can be understood by examining pairwise interactions, and second, that competition is largely hierarchical in organization. Relaxing either of these assumptions can dramatically alter intuition regarding community structure and coexistence, as noted in the extensive literature on higher‐order interactions and intransitive competition (Levine et al. 2017; Grilli et al. 2017).

Higher‐order interactions (HOIs) typically refer to cases in which pairwise interactions among species are modified by the presence of a third (or more) species in the system, and their potential importance has been acknowledged for quite some time (Vandermeer 1969; Neill 1974; Abrams 1983; Billick and Case 1994; Wootton 1994; Werner and Peacor 2003; Levine et al. 2017; Morin et al. 2022; Gibbs et al. 2022, 2024). Theoretical studies generally suggest that HOIs can promote coexistence, and under some formulations can overturn classical ideas about community diversity and stability (Bairey et al. 2016; Grilli et al. 2017). While theoretically important, there is much less known empirically about how HOIs influence coexistence, with some exceptions (e.g., Werner 1991; Peacor and Werner 1997; Buche et al. 2024). The inclusion of HOIs in statistical estimates of species interactions is suggestive of their importance in empirical systems (Mayfield and Stouffer 2017; Li et al. 2021), but they are often divorced from a mechanistic understanding of the underlying ecological processes that give rise to them (Letten and Stouffer 2019). While a more phenomenological approach to understanding HOIs may be useful in understanding some systems (Kleinhesselink et al. 2022), more mechanistic approaches that focus on the ecological processes giving rise to HOIs and how they promote coexistence in empirical communities remain less explored.

Gause's classical rule states that competitive coexistence requires sufficiently distinct niches to prevent strong competition and avoid local exclusions. While necessarily true when only two species compete, diverse communities open up the possibility of coexistence even if interspecific competition is very strong, as in the case of intransitive competition (Gilpin 1975; May and Leonard 1975; Muyinda et al. 2020). Competitive intransitivity, which emerges naturally in complex networks of interactions, is thought to provide one such mechanism for coexistence theoretically (Laird and Schamp 2006; Laird and Schamp 2008; Allesina and Levine 2011), although empirical evidence is mixed (Godoy et al. 2017; Matías et al. 2018). Often described as rock‐paper‐scissors games, a single intransitive loop can theoretically maintain an arbitrary number of species even when strongly competing (Vandermeer 2011). The actual structures of competitive networks are likely to consist of a complex of interacting intransitive and transitive components, and the details of their interaction will determine the coexistence dynamics of systems (Vandermeer 2024, 2025; Gallien et al. 2024).

There is an appreciation that empirical competitive networks are not static, but rather respond dynamically to abiotic factors in the environment (Matías et al. 2018) as well as biotic effects from within a community in the form of HOIs (Feener Jr. 2000; Chamberlain et al. 2014). Both HOIs and intransitive competition are contemporary foci of ecological theory, and they will assuredly play a part in the structure of many communities, especially in biodiverse ones. Furthermore, it seems unlikely for them to be strictly independent of one another, begging the question of how they might interact. If both HOIs and intransitivities are known to be involved in the maintenance of diversity, might these processes, under some circumstances, interact with each other to shape coexistence?

## 2 Natural History and Study System

Here, we study the competitive dynamics of a tropical ant community to understand how HOIs and intransitivities interact to structure the assembly and coexistence of the community. Ant communities provide a particularly useful model system for investigating the contingencies of competitive dynamics, as a number of non‐equilibrium processes have been proposed to influence the structure of competition and subsequent coexistence dynamics (Andersen 2008). As our model system, we focus on the ant community in a coffee‐citrus agroecosystem in the central mountains of Puerto Rico. Prior work on this ant community suggests that both HOIs and intransitive competition may be operative in the dynamics (Vandermeer and Perfecto 2023, 2024, 2025). However, direct quantification of competitive interactions, as well as the interaction between HOIs and intransitivities and their impact on coexistence, remains unknown. The HOI in this system emerges from specialist parasitoid flies in the genus *Pseudacteon* (Diptera: Phoridae) (*Pseudacteon curvatus*, *P. tricuspis* and *P. obtusus* ) which attack one of the dominant ant species in the community, *Solenopsis invicta* (Vandermeer and Perfecto 2025). These parasitoid flies are known to induce trait‐mediated effects by altering the foraging behaviour of the ants they attack (Hsieh and Perfecto 2012), reducing *S. invicta* foraging and promoting coexistence with other ant species (Orr et al. 1995; Morrison 1999). Importantly, the impact of these parasitoids on competition is thought to be non‐static as they exhibit complex spatiotemporal oscillations (Morrison et al. 1999, 2000; Morrison and Porter 2005). It has been proposed that HOIs from parasitoids may be able to decompose competitive hierarchies in ant communities to influence coexistence (Feener Jr. 2000), but the ways in which competitive networks are restructured by HOIs generally remain poorly understood (but see LeBrun 2005).

**FIGURE 1:**
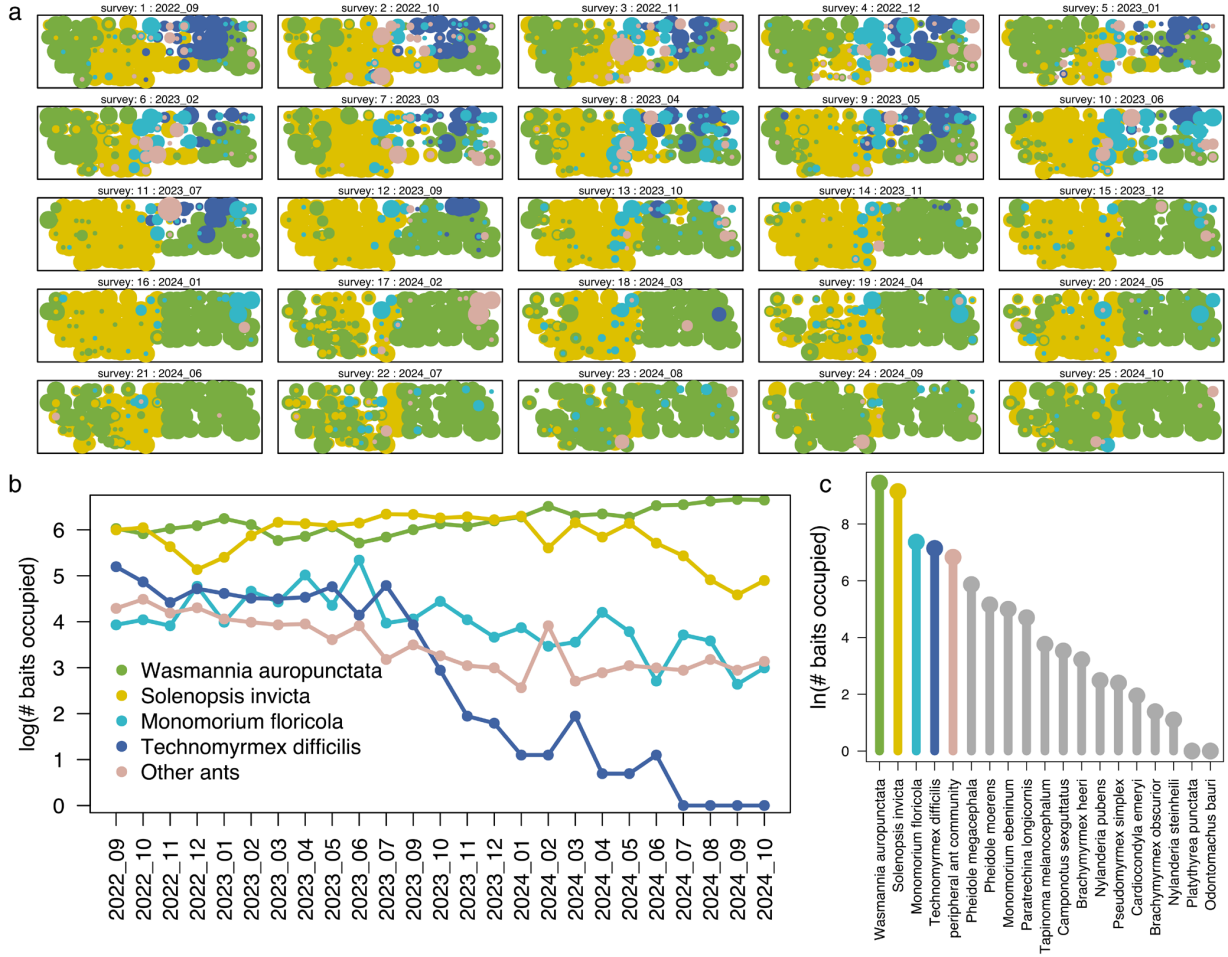
The empirical pattern of ant community dynamics in citrus trees. (a) the spatial dynamics of the ant community, with bubble sizes proportional to activity on a single tree, (b) the temporal dynamics of the dominant ants in the citrus trees from monthly surveys between September 2022 and October 2024. (c) the rank abundance distribution of the ant community spanning the duration of the surveys. Note that ‘other ants’ refers to what we call the peripheral ant community and encompasses all species less abundant than Technomyrmex difficilis.

To investigate the role of competition in structuring the ant community in our system, we systematically sampled all citrus trees within a 30‐by‐150‐m section of the coffee‐citrus farm each month for 2 years (Figure 1; see Methods). We observed 18 species during the survey, but four species are clearly predominant: *Wasmannia auropunctata* , *Solenopsis invicta* , *Monomorium floricola* and *Technomyrmex difficilis* (hereafter referred to by generic name alone) (Figure 1). In the 2 years of the surveys, we found that the spatial distribution of the ants is highly dynamic, with local extinction and colonization on trees from month to month and multi‐year oscillations in territory (Figure 1a). The spatial oscillations between the three most dominant species, *Wasmannia*, *Solenopsis* and *Monomorium*, can be seen in the time series of spatially aggregated abundances (Figure 1b), as well as the regional extirpation of the fourth most abundant species, *Technomyrmex*. Here, we leverage this empirical data to understand the drivers of the highly dynamic nature of the spatiotemporal patterns observed in this competitive community. First, we estimate pairwise competitive interactions within the community and demonstrate that, in isolation, they fail to recreate observed coexistence dynamics. We show that inclusion of an HOI, imposed by a parasitoid of a dominant species, can theoretically reorganize competitive networks to mirror the empirical dynamics of the system. We then demonstrate how temporal variation in HOIs forces the community between dominance regimes and that the interregnum between regimes is riddled with competitive intransitivities that promote coexistence.

## 3 Methods

### 3.1 Surveys of the Ant Community in Citrus Trees

Once a month, from September 2022 to October 2024, ants were surveyed on 118 citrus trees on a coffee‐citrus farm in the municipality of Utuado, Puerto Rico (UTUA2 in Perfecto and Vandermeer 2020). Five tuna fish baits were placed on each tree and checked after 5–10 and 45–60 min. The presence or absence of ant species was recorded on each bait. As noted above (Figure 1), the most abundant species on tuna baits in the system are *Wasmannia*, *Solenopsis*, *Monomorium* and *Technomyrmex*. Fourteen other species were observed on baits in the citrus trees during the survey (Figure 1c), but comprise a relatively small fraction of the baits occupied. When aggregating the number of baits occupied by all other species through time (the ‘peripheral ant community’), their abundance approximates that of *Technomyrmex*, the least common ‘dominant ant’. We focus our analysis here on these four most dominant species and treat the peripheral ant community as an aggregate to avoid missing any potentially important competitive effects of the less common species.

### 3.2 Estimating Competition Coefficients

We estimate pairwise competition between ants on the citrus trees by leveraging the two observations on the tuna fish baits at 5–10 (t_1_) and 45–60 (t_2_) minutes. We approximate the competitive effect of species *j* on species *i*, ⍺_*ij*_, as the fraction of baits on a tree where species *i* is replaced by species *j*, when species *i* was present at t_1_. At t_1_ we consider all of the baits on a tree where species *i* occurs either alone or together with species *j*, and we call this set of baits B, which contains ρ number of baits (five for all trees). We then calculate the number of baits in B where, at t_2_, species *j* is present and species *i* is absent, and call this quantity 𝛾. From this, we calculate the proportion of baits in which species *j* competitively excluded species *i*, with α_ij_ = 𝛾/ρ. We calculate α_*ij*_ for all pairwise combinations of the focal ant community for each of the 25 monthly surveys and build the matrix **A** using the average values of *⍺*_*ij*_ from all surveys as an estimate of the overall probability of pairwise competitive exclusion at the tree scale.

The column sums of **A** then give us a measure of the overall competitive effect a particular species (probability of excluding other species) has on the community and the row sums provide a measure of the overall competitive sensitivity of a particular species (probability of being excluded) in the community. Conceptually, sensitivity is the reciprocal of response competition, sensu Goldberg (Goldberg and Fleetwood 1987; Goldberg and Landa 1991). Thus, we refer to the column sum of *i* in **A** as the *effect* of *i* on the community, as it represents the sum of average probabilities of species *i* competitively excluding others in the community. We refer to the row sum of *i* in **A** as the *response* of *i*, as it is the sum of probabilities of *i* being excluded by other species in the community. The competition matrix (**A**) can be translated into a competitive outcome matrix **O**: for all *i* and *j*, where if α_*ij*_ > α_*ji*_, o_*ij*_ = 1 and o_*ji*_ = 0. This transformation creates an adjacency matrix that allows for the analysis of the network topology. The creation of competitive outcome networks assumes that even slight asymmetries in competition result in a ‘winner’ and a ‘loser’ in competition. While this represents only an approximation of competitive outcomes, it allows for structural analysis of the competitive network.

### 3.3 Direct Observation of Phorids on Baits

**FIGURE 2:**
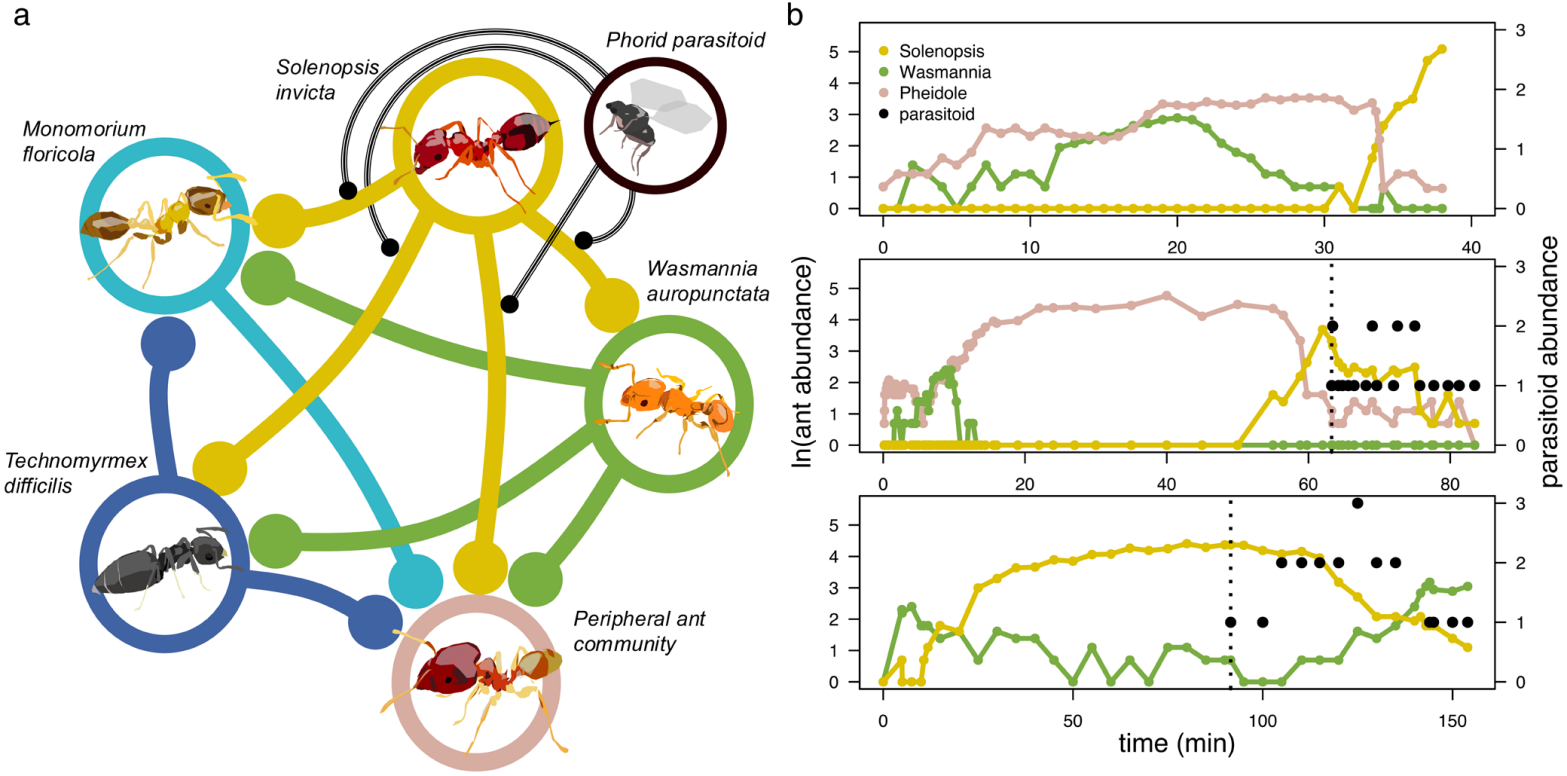
The estimated competitive structure of the ant community inhabiting citrus trees. (a) Competitive outcome network estimated from competition coefficients estimated from the time series data on tuna baits in the citrus trees, approximated for the four most dominant/abundant species in the citrus trees plus the ‘peripheral ant community’. The smaller connecting edges represent the higher‐order indirect (HOI) effect of the phorid parasitoid. (b) Time series from hotdog baits on the ground at various points in the study area, illustrating the effect of phorids on the ability of Solenopsis to compete. Top graph is only 40 min. long and illustrates how Solenopsis takes over from both Wasmannia and *Pheidole megacephala* (one of the species in the category ‘peripheral ant community’) before any phorids arrive. The middle graph illustrates how another species (in this case *P. megacephala* ) can dominate for a long time, but decline rapidly when Solenopsis arrives, yet the phorids arrive rapidly after Solenopsis swarms, thus reducing its dominance. The bottom graph illustrates Solenopsis dominating in competition with Wasmannia for an extended period (almost 2 h), but declining rapidly when the phorids finally arrive, at which point Wasmannia increases to dominate the bait again.

Observations of the dynamics of the ground ant community on baits were made to demonstrate the role of the phorid parasitoids in the control of resources. A small piece of hot dog was pinned to the center of circular notecard disks of radius 5 cm, and the site was filmed. At semi‐regular intervals, the number of ants and phorids was counted from the image. Three time series for three examples are shown to demonstrate the characteristic dynamics at baits (Figure 2b).

### 3.4 Incorporating the HOI of Solenopsis's Parasitoids

To model the HOI from phorid parasitoids on ant competition, we assume that phorids will impact both the *Solenopsis's* ability to competitively exclude other ants (effect competition), as well as its sensitivity to exclusion from other ants (response competition). In our study, we were unable to systematically measure the direct presence and impact of the phorids on the dynamics at tuna baits in the citrus trees. However, the HOI effects on competition are supported by the empirical literature on *Solenopsis* (Orr et al. 1995; Morrison 2000), detailed observations in our system (Figure 2b), as well as more broadly in ant communities (reviewed by Hsieh and Perfecto 2012). To model the HOI's impact on *Solenopsis's* competition within the community, we modify both the column and row of *Solenopsis* in the **A** matrix across a range of HOI effects from the phorids, *ω* (ranging from 1 (no HOI) to 3 (strong HOI) to capture the qualitatively distinct regimes of the community). As noted above, **A** is the matrix of estimated competition coefficients and **A**_(. , *j*)_ refers to all rows for column j, while **A**_(*j*,__.)_ refers to all columns for row *j*. For *i*, *j* = *Solenopsis*, we modify all of *Solenopsis's* competitive responses in the matrix with **A**_(*i*, .)_ = **A**_(*i*, .)_ ω^κ^ and their competitive effects in the matrix with **A**_(. , *j*)_ = **A**_(. , *j*)_ *ω* ^−κ^, where *κ* is a scaling exponent for the effect of the phorids on *Solenopsis* (held constant at 3). This approach allows us to modify the competition coefficients of *Solenopsis*, both effects and responses, as a function of the phorids' effect.

### 3.5 Spatially Explicit Model of Competition

We simulate the spatially explicit competitive dynamics of the ant community parameterized with our empirical data using a cellular automata model of competition. The structure of the model follows that of Vandermeer and Yitbarek (2012), placed on a rectangular lattice of 100 × 50 cells, corresponding to the roughly rectangular shape of the observational plot. Each cell represents a patch in space which only one species can occupy. The dynamics of occupancy in each patch are determined by the competitive interactions between species. We simulate the dynamics of a five‐species community with the competition coefficients, **A**, estimated from the survey data. The inclusion of the HOI effect of the phorid parasitoid is incorporated through the modification of **A** as described in the previous section. We first explore the static effect of the HOI on coexistence in the community, then we treat the HOI as a forced oscillator due to the known oscillatory behaviour of the phorid parasitoids. Details of the spatial competition model, its parameterization, and additional analysis of the model can be found in the Supporting Information.

## 4 Results

### 4.1 Structure of Ant Competitive Network

Our estimates of the competitive exclusion coefficients from the field data (Table S1) illustrate a strict hierarchy in the community where the peripheral ants are excluded by all species, followed by *Monomorium*, then *Technomyrmex*, then *Wasmannia*, with *Solenopsis* at the top (Figure 2a). While *Solenopsis* is clearly a dominant ant in the system, as seen by the 2 years of monthly field surveys (Figure 1), the question of how other species, and in particular *Wasmannia*, are maintained in the community naturally arises. Auxiliary data from independent sampling in the system show how the presence of *Solenopsis* parasitoids alters the outcomes of competition at baits (Figure 2b). These data show that in the short‐term (i.e., < 60 min), when *Solenopsis* arrives at a resource, it will dominate, rapidly reducing the abundance of other ants or excluding them entirely. However, when parasitoids arrive (typically after more than 60 min), there is an abrupt drop in *Solenopsis*'s abundance, allowing for subsequent increases in competitors at baits. These data, as well as behavioural observations in this system, suggest that the HOI from *Solenopsis's* parasitoids directly influences the pairwise probabilities of competitive exclusion, by simultaneously reducing *Solenopsis's* ability to exclude other ants and increasing its susceptibility to exclusion. It further suggests that measurements of competitive exclusion at relatively short time scales (< 60 min) capture only a fraction of the elements that contribute to the maintenance of coexistence.

### 4.2 HOIs Promote Intransitivities Between Competitive Regimes

**FIGURE 3:**
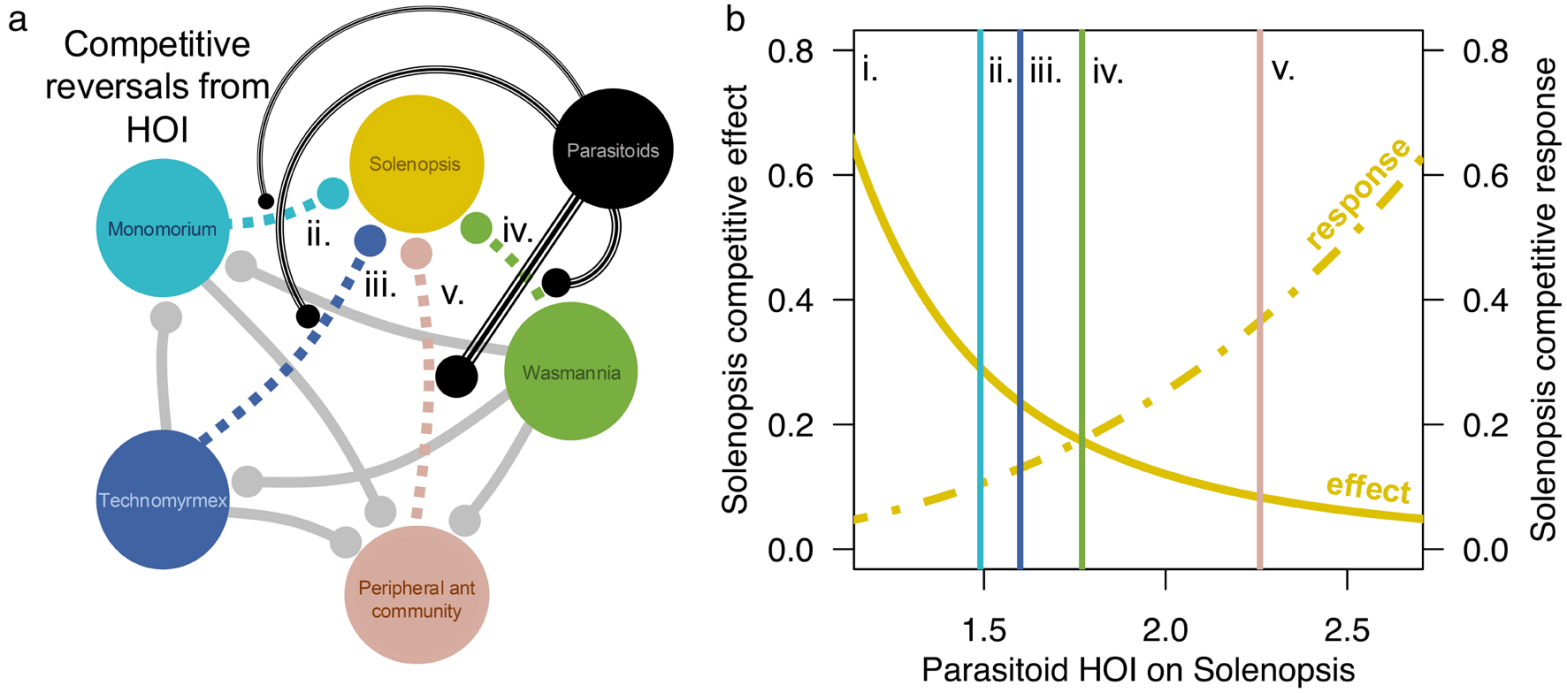
The higher order interaction (HOI) from the phorid parasitoid restructures community interactions by modifying Solenopsis's effect and response competition with other ants in the community. (a) graphical depiction of how the phorid HOI reverses outcomes in a competitive network. The size of the striped‐black lines illustrates the relative strength of the HOI necessary to reverse Solenopsis's ability to competitively exclude other ants in pairwise competition (dashed lines). (b) Illustrates how the strength of the HOI reduces Solenopsis's effect competition (i.e., sum of probabilities of Solenopsis excluding other ants in the community) (solid yellow line), and increases Solenopsis's response competition (i.e., sum of probabilities of Solenopsis being excluded by other ants in community) (dashed yellow line). Vertical solid lines illustrate the critical value of HOI from the phorid parasitoid necessary to reverse the outcome of pairwise competition with Solenopsis. Teal corresponds to Monomorium, navy blue to Technomyrmex, green to Wasmannia, and peach to the peripheral ant community. Roman numerals on a and b highlight which competitive outcome is reversed by the parasitoid HOI.

Given the known importance of parasitoids in restructuring competition in this system and in the broader literature, we explore how the HOI from parasitoids can reshape the topology of the competitive network in this ant community. We identify the critical values of the HOI that result in competitive reversals between *Solenopsis* and other species in the community (Figure 3). The vertical lines in Figure 3b show the values of HOI where a given ant will win in competition against *Solenopsis* based on the competitive outcome matrix. As the strength of the HOI increases, the first pairwise outcome to flip is with *Monomorium* (Figure 3bii), followed by *Technomyrmex* (Figure 3biii), *Wasmannia* (Figure 3biv), and then finally with the peripheral ant community (Figure 3bv). Note that the order of competitive reversals is not directly related to the hierarchy of predicted competitive outcomes (Figure 2a), something which is likely an emergent feature of asymmetries in the matrix of pairwise competitive effects (see Supporting Information for details and discussion).

**FIGURE 4:**
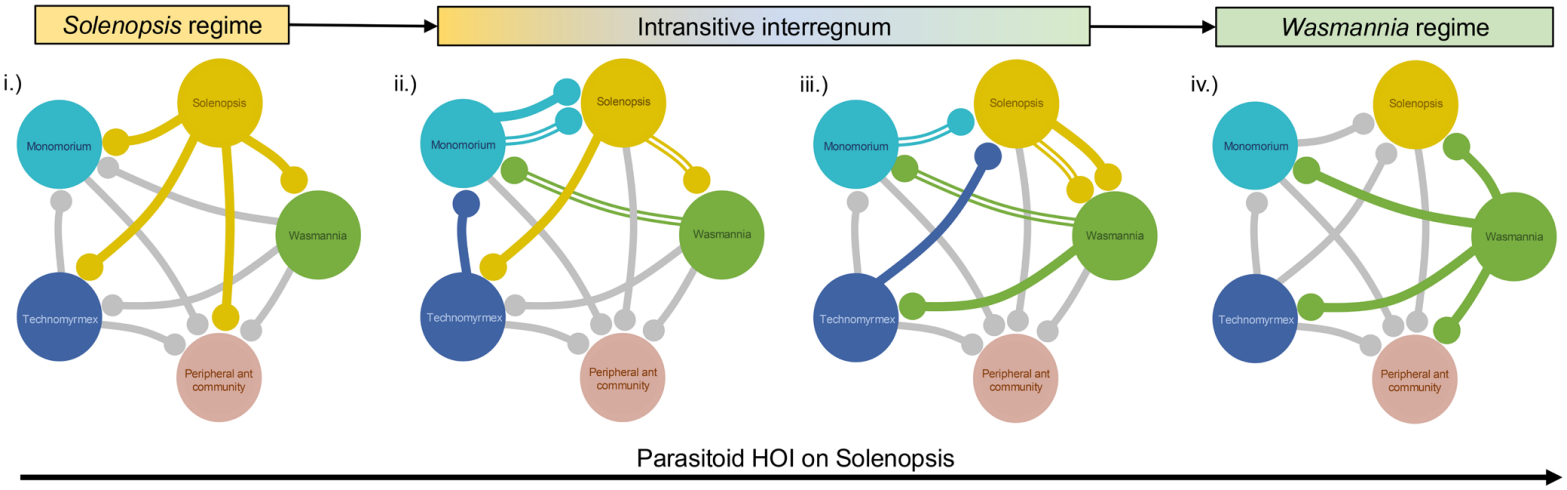
The competitive network as rewired from the higher‐order interaction (HOI) effect from phorid parasitoids on Solenopsis. The Roman numerals correspond to the competitive reversals associated with increasing HOI shown in Figure 3. The coloured lines highlight structural changes in the competitive network. The different coloured line types (hollow and solid) illustrate distinct intransitive loops. Note that four‐species intransitive loops in (ii) and (iii) are not illustrated in the figure. With increasing HOI from phorids we find (i) a competitive hierarchy with Solenopsis on top, (ii) competitive reversal with Monomorium creates two intransitive triplets, (iii) competitive reversal with Technomyrmex results in loss of one intransitive triplet and creation of another, (iv) competitive reversal with Wasmannia results in competitive hierarchy with Wasmannia on top. Note that (v) from Figure 3 is absent, but also ends with a competitive hierarchy with Solenopsis on the bottom instead of the peripheral ant community.

Consideration of the parasitoid HOI on *Solenopsis* predicts two distinct regimes at the extremes of the parasitoid HOI effect. When the effect of the HOI is weak or absent, we expect the dominance hierarchy as previously described, with *Solenopsis* competitively excluding all other ants in the community (Figure 4i). At the other extreme, when the HOI from the parasitoid is strong, we predict that *Wasmannia* will competitively exclude all other ants in the community in a dominance hierarchy (Figure 4iv). These two competitive dominance regimes illustrate a complete restructuring of the competitive network and point towards the importance of the two most dominant species (Figure 1). Interestingly, across a continuum of the HOI we see the emergence of complex competitive networks in the interregnum between the two dominance regimes, where multiple interacting intransitive loops occur (Figure 4ii,iii).

When the HOI flips the competitive outcome between *Solenopsis* and *Monomorium* we find the onset of three distinct and interacting intransitive loops. First, we observe a *Solenopsis*‐*Wasmannia*‐*Monomorium* intransitive triplet, a *Solenopsis*‐*Technomyrmex*‐*Monomorium* triplet, and a *Solenopsis*‐*Wasmannia*‐*Monomorium‐Technomyrmex* four‐species intransitive loop (Figure 4ii). Increasing the strength of the HOI, we find the competitive outcome between *Solenopsis* and *Technomyrmex* flips. While the *Solenopsis*‐*Wasmannia*‐*Monomorium* and four‐species intransitive loops remain in the network, a new triplet of *Solenopsis*‐*Wasmannia*‐*Technomyrmex* emerges (Figure 4iii). Further increasing the strength of the HOI results in the emergence of the alternative regime of *Wasmannia* at the top of the competitive hierarchy (Figure 4iv). The final competitive reversal between *Solenopsis* and the peripheral ant community (Figure 3bv) maintains the same network topology, but with *Solenopsis* at the bottom of the hierarchy (not illustrated in Figure 4). Notably, the previously suggested intransitive triplet of *Solenopsis*‐*Wasmannia*‐*Monomorium* thought to be operative in the coffee layer of the system (Vandermeer and Perfecto 2023) is maintained in the interregnum between the *Solenopsis* and *Wasmannia* regimes.

### 4.3 Spatially Explicit Dynamics of Competitive Coexistence

**FIGURE 5:**
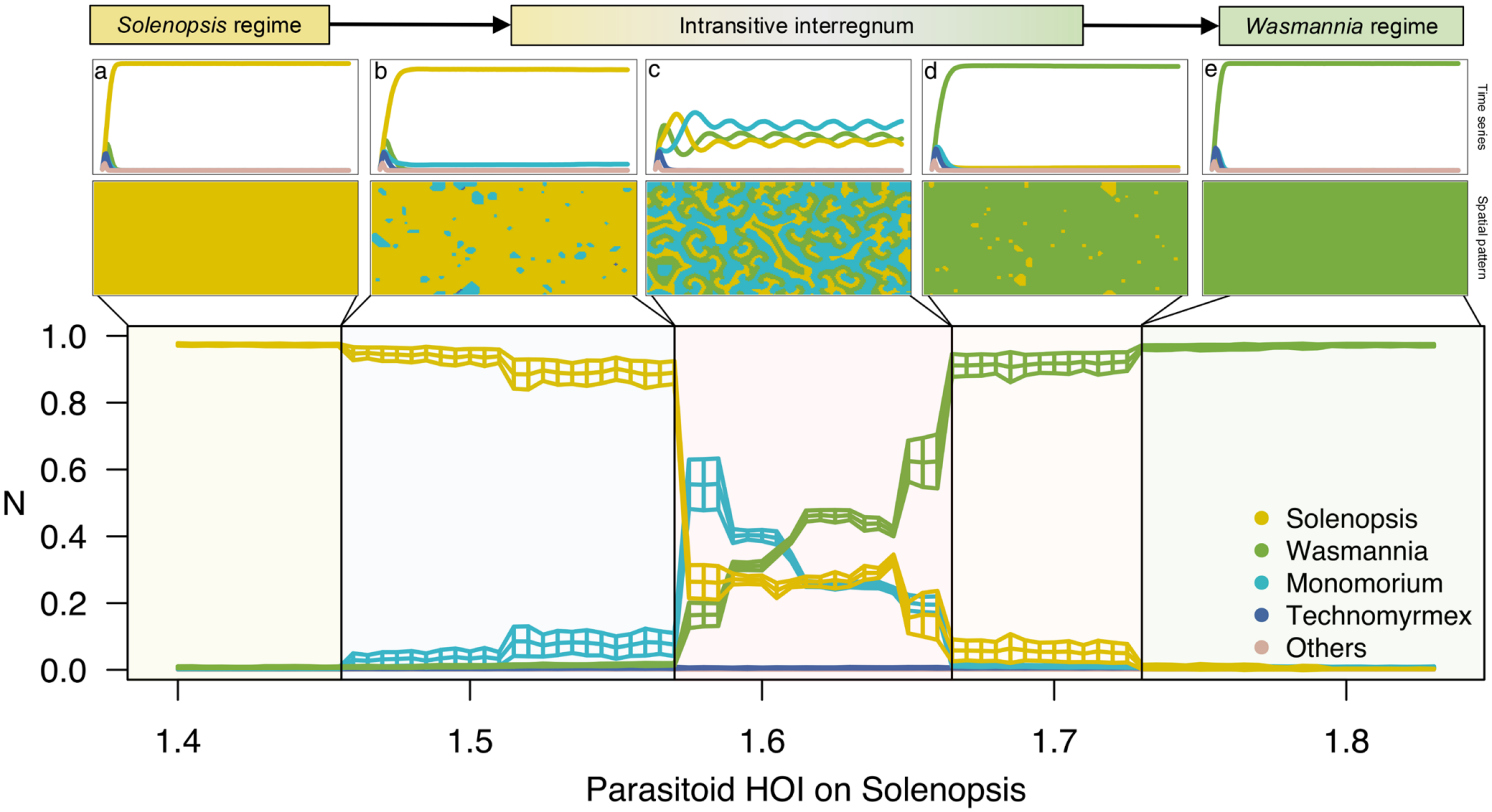
The model spatial dynamics of increasing HOI strength on the community structure of the citrus ant community. The colours on the main plot correspond to the dominant species composition from the parameter combination, yellow: Solenopsis dominance, blue: Solenopsis‐Monomorium, red: Solenopsis‐Monomorium‐Wasmannia, orange: Wasmannia‐Solenopsis, and green: Wasmannia dominance. Top plots show the time series and spatial patterning with the dominant interaction structure above them. Bottom plot shows the average proportion of the landscape occupied by each species along with the standard deviation from 100 simulations.

Simulating the spatial dynamics of competition, we find that although the two distinct regimes of *Solenopsis* dominance and *Wasmannia* dominance are present for extreme values of the HOI, the interregnum dynamics between these two regimes are distinct from what is predicted from the competitive outcome matrix alone (Figure 5). When the HOI effect is strong enough for the competitive reversal between *Monomorium* and *Solenopsis*, *Monomorium* is able to successfully invade the plot in patches embedded in a matrix of *Solenopsis* (Figure 5b). The predicted intransitive loops from the competitive outcome network (Figure 4) are notably not established for this value of HOI. Increasing the HOI further results in the perpetual establishment of the *Solenopsis‐Wasmannia‐Monomorium* intransitive triplet, where all three species oscillate perpetually and coexist (Figure 5c). Further increasing the HOI effect results in the loss of *Monomorium* from the system, with *Wasmannia* dominating except for some clusters of *Solenopsis* (Figure 5d). Finally, when the HOI is very strong, *Wasmannia* takes over the lattice completely (Figure 5e).

Analysis of the competitive networks and model of spatial competition both show that two competitive hierarchical regimes exist when the HOI is either relatively weak (*Solenopsis* regime) or relatively strong (*Wasmannia* regime), with intransitive structures predominant in the interregnum between the two regimes. As previously mentioned, the effect of the HOI from the phorid parasitoids is not expected to be constant as their populations exhibit complex oscillations over time (Morrison et al. 2000; Morrison and Porter 2005). Next, we explore how a variety of oscillations in HOI influence the community assembly and coexistence dynamics of the community. Across a broad range of amplitude and frequency of HOI, *Solenopsis* and *Wasmannia* are the two most abundant species, with *Monomorium* being the third. This is the general pattern observed in empirical data (Figure 1) and is suggestive of oscillations in HOI playing a role in the community assembly of this ant community.

**FIGURE 6:**
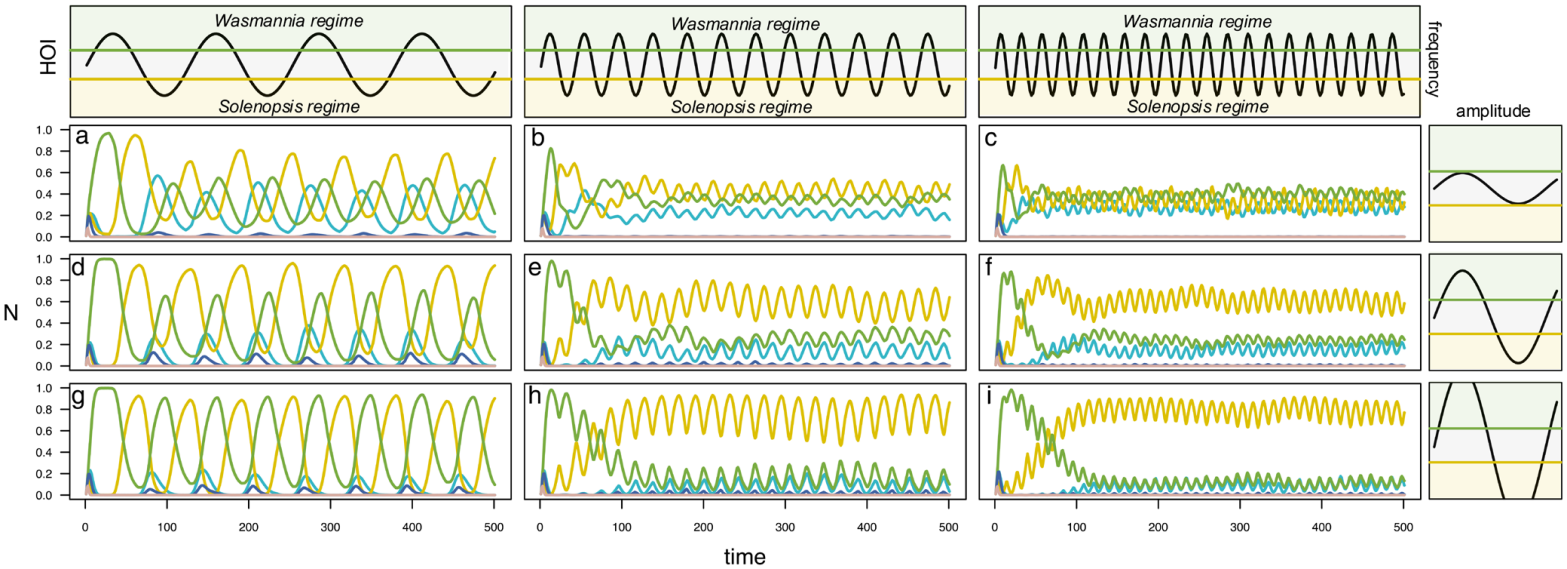
Temporal heterogeneity in competitive dynamics determines coexistence of the ant community. The columns show oscillations of varying frequency of the HOI from phorids and the rows show varying amplitudes. Time series show an average of 100 replicate simulations for a given parameter combination of frequency and amplitude. When the HOI moves below the yellow line Solenopsis is the long‐term winner in competition (yellow area), when the HOI moves above the green line Wasmannia is the long‐term winner in competition (green area), and the interregnum between regimes is between the two lines (grey area). A single amplitude is shown on the columns illustrating frequency and a single frequency is shown on the rows illustrating amplitude. Colours correspond to dynamics of the ants in the community with Solenopsis in yellow, Wasmannia in green, Monomorium in teal, and Technomyrmex in navy blue.

When the amplitude of HOI oscillations is bound within the interregnum, the *Solenopsis‐Wasmannia‐Monomorium* intransitive loop dominates, and the relative abundances of the three species become more uniform as the frequency of oscillations increases (Figure 6a–c). As the amplitude of the HOI oscillations increases, *Solenopsis* dominates when the frequency is high (Figure 6f,i), but when the frequency is low, we observe the periodic invasion of *Technomyrmex* (Figure 6d,g). Similar periodic emergence and extirpation of *Technomyrmex* is observed on our survey plot (Figure 1a) and is notably absent when the HOI is static (Figure 5). A more detailed analysis of the model is presented in the Supporting Information, but the general results here suggest that variability in oscillations of the HOI has potentially important implications for patterns of community assembly and coexistence in this system.

## 5 Discussion

Using 2 years of monthly survey data, we estimate the strength of competitive interactions among the various species in an arboreal ant system to construct competitive networks and parameterize a model of spatial competition. We find that in the absence of the higher‐order effect from a parasitoid on the dominant ant in the system, our data predict monodominance of a single ant, *Solenopsis invicta* . It appears that a higher‐order interaction effect from the parasitoid is a key component of the system, maintaining the system's diversity by reshaping the overall competitive network. We find that at either extreme of the parasitoid's effect on the ant, there exist distinct competitive hierarchies (regimes) with each of the two most common species at the top. When the HOI of the parasitoid falls between these two regimes, the parasitoid promotes multiple interacting competitive intransitive loops in the community. We suggest that temporal variation in this HOI effect from the parasitoid may act to maintain diversity as the system oscillates between competitive hierarchies, promoting the dynamic rewiring of competitive interactions that create biodiversity‐stabilizing intransitivities.

The notion of a regime and its transformation more generally has become a major subject in ecology (Scheffer et al. 2001). Typically, slow transformations of ecosystems, such as plant succession or eutrophication, were commonly cited in early ecological narratives (e.g., Tansley 1935; Lindeman 1942). Here, we suggest that an HOI may be a factor in regime change—a shift from dominance of one species to another. Furthermore, when more than two competing species are involved, the regime shift may involve considerable complexity in the community assembly process. Most notably, we find the interregnum between two dominance regimes transitions through a series of complex competitive structures, which include multiple intransitivities, driven by HOIs. The dynamics within the interregnum between regimes highlight the importance of non‐equilibrium dynamics in shaping coexistence.

While two species ( *Solenopsis invicta* and *Wasmannia auropunctata* ) dominate the surveyed area (as they frequently do in many regions throughout the central mountains of Puerto Rico (Perfecto and Vandermeer 2020)), at any one site of relatively small size, only one of the two will be found to predominate (Figure 1). While there are other species in the system, their occurrence depends on the details of the interaction between the two dominant species. Both field observations/measurements and forays into theory strongly suggest that a complex of parasitoid phorid flies elicit a behavioural response in the dominant competitor (*Solenopsis*) (Vandermeer and Perfecto 2024, 2025), thereby reducing and even reversing its dominance, a clear HOI (Figure 2). Our work here supports the existence of two competitive dominance regimes, at one extreme (no‐HOI) with *Solenopsis* at the top of the hierarchy, and the other (strong‐HOI) with *Wasmannia* at the top of the hierarchy, where the transition from the *Solenopsis* to *Wasmannia* regime is induced by the HOI from specialist parasitoids of *Solenopsis*. Furthermore, it is in the space between these regimes, the interregnum, where other species in the community are able to coexist. This interregnum arises from the effective reduction in the generalized competitive ability of *Solenopsis* due to the HOI, as well as the emergent intransitive structures in the community. This theoretical framing and the direct observations that led to it suggest that two general ecological phenomena, HOIs and intransitive competition, can be directly related to each other.

Intransitive competition has been suggested as an important mechanism influencing the diversity of ecological communities both theoretically (Laird and Schamp 2006; Vandermeer and Yitbarek 2012) and empirically (Kerr et al. 2002; Soliveres et al. 2015; Soliveres and Allan 2018). Intransitivities allow for the coexistence of species involved in the intransitive loop itself (Gilpin 1975; May and Leonard 1975), but also may act as a keystone structure to maintain species that might be indirectly linked in the competitive network (Grilli 2023; Vandermeer and Perfecto 2023). While the role of intransitive competition in maintaining diversity is relatively well understood theoretically (e.g., Allesina and Levine 2011; Schreiber and Killingback 2013; Gallien et al. 2017; Muyinda et al. 2020; Vandermeer 2024), there is comparatively little empirical work evaluating its potential importance, often with contrasting findings. In one study (Godoy et al. 2017), intransitive structures were reported to be relatively rare in an 18‐species plant community and, when present, were deemed unimportant in promoting the coexistence of other species. In contrast, a synthesis from Soliveres et al. (2015) found that intransitivity appeared in a majority of plant communities studied and is correlated with increased species richness. Our study here suggests that intransitivities likely play a role in promoting diversity in this tropical ant community, something suggested but not tested in previous studies of the same system (Vandermeer and Perfecto 2023, 2024, 2025).

Similar to intransitive competition, the potential importance of HOIs in structuring competitive community dynamics has long been acknowledged (Vandermeer 1969; Neill 1974; Abrams 1983; Billick and Case 1994; Werner and Peacor 2003; Levine et al. 2017; Morin et al. 2022). There have been various attempts to categorize interactions that fall under the very general umbrella of “higher order.” Some studies distinguish between HOIs that emerge in trophic (Terry et al. 2017) versus competitive communities (Gibbs et al. 2024), others distinguish between behavioural and consumptive (Orrock et al. 2008), yet others distinguish between density‐mediated and trait‐mediated (Werner and Peacor 2003). The situation here is clear from direct observations: that the parasitoid modifies the behaviour of a dominant competitor in such a way that its competitive effectiveness is reduced (see Figure 2 and Orr et al. 1995). Although the mechanistic incorporation of HOIs into ecological models varies among theoretical studies, it is often suggested that there is a diversity‐promoting effect of HOIs in communities (Bairey et al. 2016; Grilli et al. 2017), though the structure of HOIs is also an important determinant of coexistence (Gibbs et al. 2022; Gibbs et al. 2024). Here we find that HOIs can promote coexistence, but only under certain parameter regimes; where if the HOI effect is either too strong or too weak, it reduces the diversity of the system as it collapses to one of two regimes (Figure 5). Based on simple qualitative reasoning, the existence of two regimes is to be expected at the extremes of competition, and, in a community context, it is not surprising that between those regimes other species can coexist, highlighting the central role of oscillations in HOI (Figure 6).

We suggest that the maintenance of diversity between these two regimes may be partially determined by the temporal variability of the parasitoid and its effect on competitive interactions in the community. We found that the nature of these oscillations, particularly their frequency (Figure 6), is critical in explaining community dynamics. While we only have preliminary data on parasitoid oscillations (Supporting Information; Figure S2), prior work on parasitoids of *Solenopsis* shows that complex oscillations tend to be the norm, where both frequency and amplitude of oscillations vary through time and across monitored sites (Morrison et al. 1999; Morrison et al. 2000; Folgarait et al. 2003; Morrison and Porter 2005; Henne and Johnson 2009). The underlying mechanism behind these oscillations is likely due to a variety of factors, including the oscillatory nature of consumer‐resource interactions, as well as environmental forcings. It has been suggested that seasonal forcing in the form of rainfall may be an important determinant of oscillations in parasitoid abundance (Morrison et al. 1999; Henne and Johnson 2009), but published data clearly exhibit complex oscillations, suggesting that environmental forcing is not the sole determinant. Complex multi‐peaked power spectra are known to emerge from the forcing of oscillators (e.g., seasonal forcing of consumer‐resource systems) (Pascual and Ellner 2000; Vandermeer et al. 2001). Given the absence of a clearly known mechanism behind these oscillations in these systems, we explored a range of scenarios here to understand the general impact of oscillating phorids/HOIs on structuring community dynamics and coexistence. Disentangling the spatiotemporal drivers of phorid population dynamics is an important next step towards understanding coexistence in ant communities.

As this study highlights, the dynamics of competition are likely context dependent, being influenced by spatial and temporal factors including HOIs. While it is appreciated that HOIs from parasitoids can restructure competitive interactions in ant communities (Feener Jr. 2000; Andersen 2008), it has also been shown that this effect is context‐dependent on the types of resources that are the focus of competition (Morrison et al. 2000; LeBrun 2005). In this study, we quantified interactions on tuna baits, which only represent a single type of high‐value resource that is relatively large and immobile with high lipid and protein content. The natural history and foraging dynamics of the ant species in this community will have important implications for our observed competitive dynamics. For example, *Solenopsis* frequently swarms and completely covers large resources (Wang et al. 2018) while *Monomorium* swarms and defends baits with ant‐repellent alkaloids (Jones et al. 1982). Both of these strategies may favour control over large resources and produce priority effects which are known to be operative in ant communities (Levins et al. 1973; Torres 1984). It is likely that a unique matrix of pairwise competitive effects exists for a variety of resource types within this community, and that features of the resource, such as nutritional content (e.g., carbohydrates, lipids, proteins) and size (e.g., small movable insect versus large animal carcass) will provide a different picture of competitive dynamics.

This work highlights how HOIs can mechanistically reshape competitive dynamics of communities and can decompose competitive hierarchies into intransitive modules that are important for species coexistence. The context dependence of competitive outcomes in our empirical system likely emerges from both spatial and temporal variation in HOIs and suggests that analysis of static competitive interactions likely represents only a temporary snapshot of community structure, leaving obscure the true underlying complexity of competitive interactions. Similar temporal variation in interaction networks is well appreciated for some systems, such as plant‐pollinator interactions (CaraDonna et al. 2017; Zoller et al. 2023), and there is growing appreciation of its potential importance in competitive interactions (Rudolf 2019; Yang 2020; Yin and Rudolf 2024). Although relatively few studies to date have explored the significance of temporal variation in HOIs through time (but see Hoverman and Relyea 2008; Zou et al. 2024), this study suggests they may play a critical role in both community assembly and coexistence.

## Author Contributions

All authors designed the study. Data were collected by all authors. Data analysis, visualization, and modelling was done by Zachary Hajian‐Forooshani with input from John Vandermeer and Ivette Perfecto. Zachary Hajian‐Forooshani wrote the first draft of the manuscript and John Vandermeer and Ivette Perfecto contributed to revisions.

## Supporting information


**Table S1:** Matrix of the estimated competition coefficients **A**. The competitive effect of species *j* on species *i*, *⍺*_*ij*_, is the conditional probability that species i is replaced by species *j*, conditional that species *i* was present at the first check (see Methods). Community response is the row sum of **A** representing the overall sensitivity of the species to competitive exclusion from the community. Community effect is the column sum of A and represents the overall ability of a species to competitively exclude others in the community.
**Figure S1:** Effect and response competition of the community. Each point represents the row and column sums for each species in the community. Response competition refers to the ability of other species in the community to exclude a focal species while the effect competition refers to the ability of the focal species to competitively exclude others in the community.
**Figure S2:** (A) Phorid abundance in three parts of the plot that correspond to Solenopsis dominance (red), Wasmannia dominance (green) and a contested territory between these two species. (B) Time series of the plot for 25 monthly surveys showing the dominant ant species present (yellow = Solenopsis, Green = Wasmannia, teal = Monomorium, navy = Technomyrmex).
**Figure S3:** Immigrations as calculated from the empirical data. The solid lines show the variation through time and the dashed lines show the average value which was used for the simulations.
**Figure S4:** Illustrates the parameters of the forced HOI oscillations in the model. Solid green line shows the approximate value of HOI for the onset of the Wasmannia regime, while solid yellow line shows the approximate value of HOI for the onset of the Solenopsis regime. *V*_offset_ shows the center point of the HOI function which falls at the center of the interregnum (space between Wasmannia and Solenopsis regimes). f shows the frequency, and A the amplitude of the HOI oscillations.
**Figure S5:** Single replicates from the parameter combinations used in Figure 6 in the main body.
**Figure S6:** Time series from the competition model with wider range of frequency and amplitude in HOI oscillation. Columns show different values of frequency and rows amplitude.
**Figure S7:** Rank abundance distributions emerging from the model. Different columns show various frequencies of HOI oscillations and different rows show various amplitudes.

## Data Availability

All data and code used in the study can be found at https://figshare.com/s/13cf6595c5560849fd59.
