# Supplementary material for: Higher‐Order Interactions Can Promote Coexistence by Rewiring Intransitivities Into Competitive Networks
Source: Ecol Lett. 2026 Jun 30;29(7):e70415. doi: 10.1111/ele.70415 (PMC13317470; doi:10.1111/ele.70415)
Supplement: Supplementary file 1 — Table S1: Matrix of the estimated competition coefficients A. The competitive effect of species j on species i, ⍺ ij , is the conditional probability that species i is replaced by species j, conditional that species i was present at the first check (see Methods). Community response is the row sum of A representing the overall sensitivity of the species to competitive exclusion from the community. Community effect is the column sum of A and represents the overall ability of a species to competitively exclude others in the community. Figure S1: Effect and response competition of the community. Each point represents the row and column sums for each species in the community. Response competition refers to the ability of other species in the community to exclude a focal species while the effect competition refers to the ability of the focal species to competitively exclude others in the community. Figure S2: (A) Phorid abundance in three parts of the plot that correspond to Solenopsis dominance (red), Wasmannia dominance (green) and a contested territory between these two species. (B) Time series of the plot for 25 monthly surveys showing the dominant ant species present (yellow = Solenopsis, Green = Wasmannia, teal = Monomorium, navy = Technomyrmex). Figure S3: Immigrations as calculated from the empirical data. The solid lines show the variation through time and the dashed lines show the average value which was used for the simulations. Figure S4: Illustrates the parameters of the forced HOI oscillations in the model. Solid green line shows the approximate value of HOI for the onset of the Wasmannia regime, while solid yellow line shows the approximate value of HOI for the onset of the Solenopsis regime. V offset shows the center point of the HOI function which falls at the center of the interregnum (space between Wasmannia and Solenopsis regimes). f shows the frequency, and A the amplitude of the HOI oscillations. Figure S5: Single replicates from the parameter combin [file ELE-29-0-s001.docx]

**Supplementary material**

Higher-order interactions can promote coexistence by rewiring intransitivities into competitive networks

Zachary Hajian-Forooshani^*^ Ivette Perfecto Warren Irizarry and John Vandermeer

*Correspondence: zachary.hajian-forooshani@idiv.de

**Sections**

1. Competitive structure of citrus ant community

*i. Estimation and structure*

*ii. HOI rewiring of competitive network*

2. Dynamics of Solenopsis’s phorid parasitoids

3. Modeling competitive dynamics of the ant community

*i. Model description*

*ii. Parametrization and initialization*

*iii. Model dynamics*

**1. Competitive structure of citrus ant community**

*Estimation and structure*

As mentioned in the main body of the text, we calculated the average proportion of pairwise bait exclusions on citrus trees as a measure of competition between species in the system. Takeover, or exclusion, on a bait was measured by checking each one of the five tuna baits on a citrus tree at approximately 5-10 minutes (t_1_) after setting and then at approximately 45-60 minutes (t_2_). We approximate the competitive effect of species *j* on species *i*, ⍺*_ij_*, as the fraction of baits on a tree where species *i* is replaced by species *j*, when species *i* was present at t_1_. At t_1_ we consider all of the baits on a tree where species *i* occurs either alone or together with species *j*, and we call this set of baits B, which contains ρ number of baits (five for all trees). We then calculate the number of baits in B where, at t_2_, species *j* is present and species *i* is absent, and call this quantity 𝛾. From this, we calculate the proportion of baits in which species *j* competitively excluded species *i,* with $\alpha$_ij =_ 𝛾/ρ. We calculate $\alpha$_ij_ for all pairwise combinations of the focal ant community for each of the 25 monthly surveys and build the matrix **A** using the average values of ⍺_ij_ from all surveys as an estimate of the overall probability of pairwise competitive exclusion on a tree.


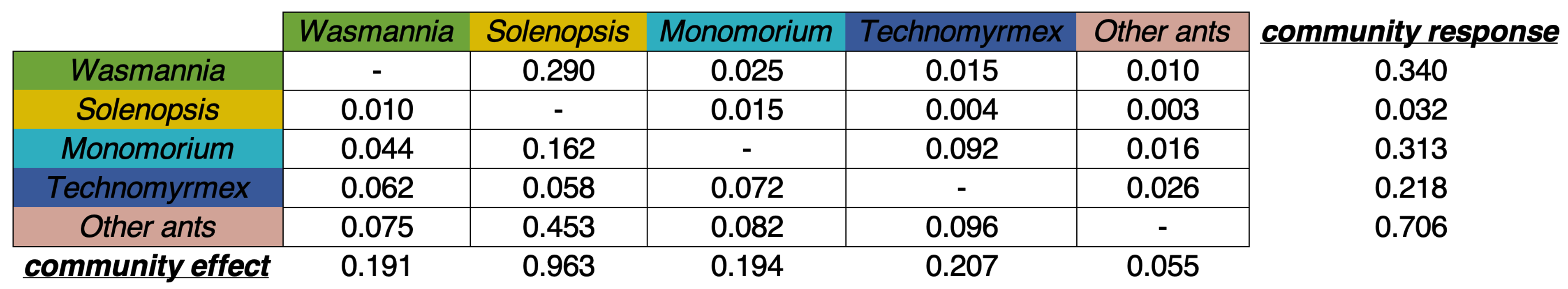


*Table S1. Matrix of the estimated competition coefficients* ***A****. The competitive effect of species j on species i, ⍺_ij,_ is the conditional probability that species i is replaced by species j, conditional that species i was present at the first check (see Methods). Community response is the row sum of* ***A*** *representing the overall sensitivity of the species to competitive exclusion from the community. Community effect is the column sum of* ***A*** *and represents the overall ability of a species to competitively exclude others in the community.*

The general competitive structure of the community can be understood through the sums of effect and response competition in the matrix of competition coefficients **A** (Table S1). The column sums of the matrix represent the total competitive effect that a species has on the community (effect competition) and the row sums represent the total competitive response of the species to the community (response competition), which represents its sensitivity to the community. Plotting the effect and response competition of each species in the community, there is a clear trade-off in the community (Fig. S1). We see that *Solenopsis* which has the largest effect on the community has the smallest response, meaning that it is best at competitively excluding other species in the community, but is very resistant to competitive exclusion itself. The peripheral ant community is at the other extreme with the lowest effect competition and high response. The other abundant members of the community fall between *Solenopsis* and the other ants in the community, and occur near the 1:1 line but trend towards higher responses than effects. This highlights the very “unbalanced” nature of competition in the community where *Solenopsis* is on top of a competitive hierarchy.


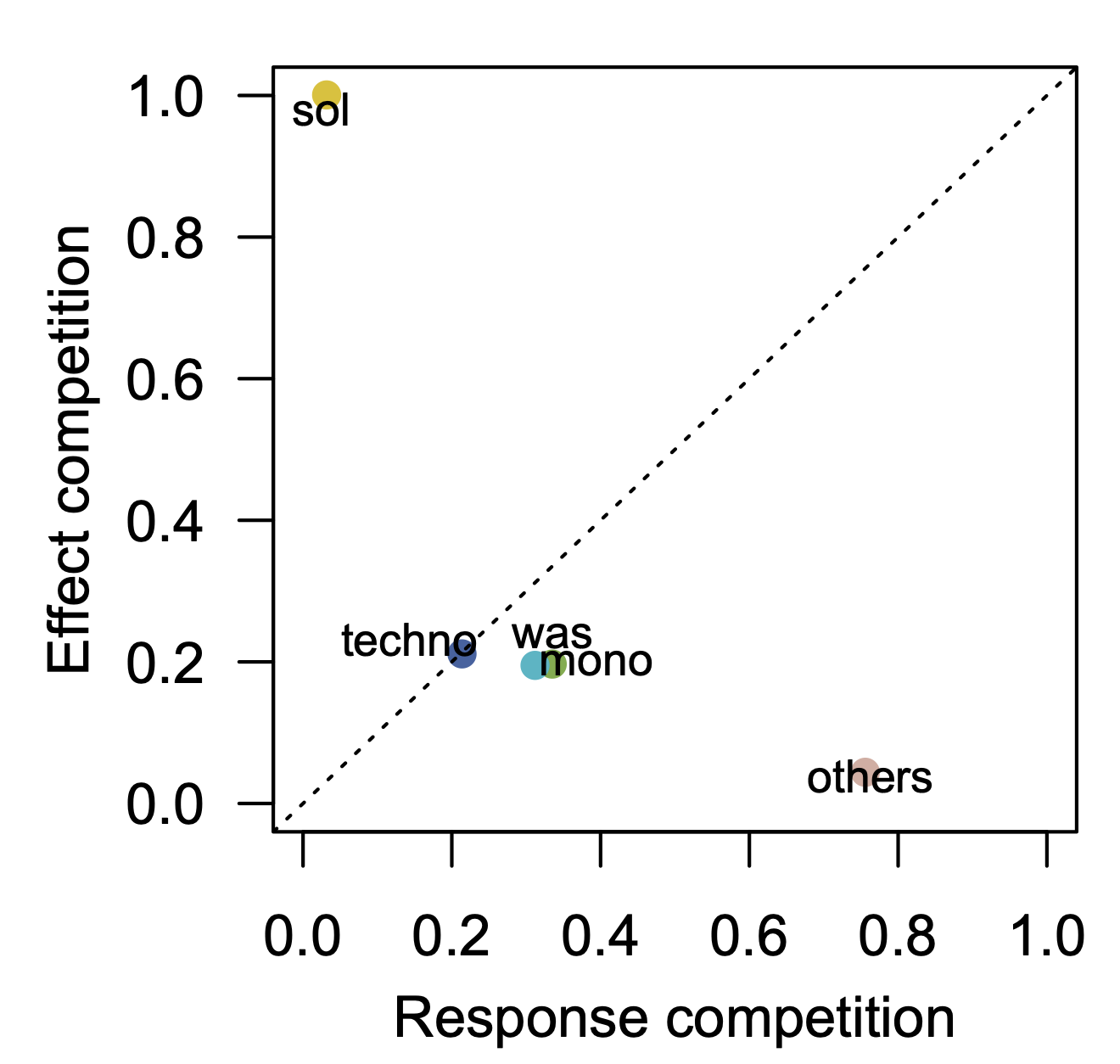


*Figure S1. Effect and response competition of the community. Each point represents the row and column sums for each species in the community. Response competition refers to the ability of other species in the community to exclude a focal species while the effect competition refers to the ability of the focal species to competitively exclude others in the community.*

*HOI rewiring of competitive network*

We note in the results of the main body of the manuscript that the order in which the HOI reverses pairwise competitive outcomes does not correspond to the hierarchical structure of the community. It is perhaps reasonable to expect that since *Wasmannia* is the second most dominant species (i.e., it wins in competition with all species except *Solenopsis*) that it should be the first species to flip its competitive outcome and beat *Solenopsis* right away as the strength of the HOI impact on *Solenopsis* competition coefficients increases. However, we find that it is the 3^rd^ species to exhibit the competitive reversal, after *Monomorium* and *Technomyrmex* (Figure 3 in main body). This is seemingly due to asymmetry in the structure of competition as seen in the matrix, **A** (Table S1), as well as how it is translated to the competitive outcome matrix, **O**. We translate the coefficients from **A** to binary outcomes of competition in a new adjacency matrix, **O**, by making pairwise comparisons between coefficients in A to determine an estimated winner in competition. Every entry in the matrix, **A**, is translated to a new matrix, **O**, where if **A**[*i,j*] > **A**[*j,i*] then **O**[*i,j*] is set equal to 1 and **O**[*j,i*] is set equal to 0. This implies that if species *j* is more likely to exclude *i* than *i* is to exclude *j* then *j* wins in competition, thus determining the competitive outcome.

By looking at the entries of **A**, we see that the difference between **A**[W, S] and **A**[S, W] is much larger than for any other pair except for **A**[Ot, S] and **A**[S, Ot] (W = Wasmannia, S=Solenopsis, Ot=other ants). This asymmetry between *Wasmannia* and *Solenopsis* means that although *Wasmannia* is second in the hierarchy and can exclude other ants in the community, it loses to *Solenopsis* by a larger value than it does to either *Monomorium* or *Technomyrmex*. *Wasmannia’s* strong response to and weak effect on *Solenopsis* means that its flip in competitive outcome with *Solenopsis* occurs after those of *Monomorium* and *Technomyrmex*, even though *Wasmannia* wins pairwise competitions with them. Thus, it is this asymmetry in the competitive effects that creates the interregnum between *Solenopsis* and *Wasmannia* regimes.

**2. Dynamics of *Solenopsis’s* phorid parasitoids**

Sites were established after the sampling dates presented in the main body of the manuscript to look at the dynamics of the phorids through time. Similar to other studies (e.g., Morrison et al., 1999), we find that the phorid populations are oscillatory through time. Data was collected with phorid traps which were placed on the ground near *Solenopsis* nests approximately every other week from March 12, 2025 to July 16, 2025. Interestingly, we find that sites where *Solenopsis* dominated during our surveys (left side of the plot) are sites where phorids are practically absent from the traps (Fig. S2). On the other hand, we find that sites where *Wasmannia* dominates on the plot, are sites where phorids are very abundant on the traps (Fig. S2). Furthermore, in the middle of the plot, which is largely contested territory between *Solenopsis* and *Wasmannia*, we find an intermediate amount of phorids (Fig. S2). This data shows that the locations of phorids are associated with competitive outcomes in the system, and that the population dynamics of the phorids are oscillatory. Thus, it is likely an interaction between the oscillations and spatial heterogeneity that shape the overall spatiotemporal dynamics of the community.


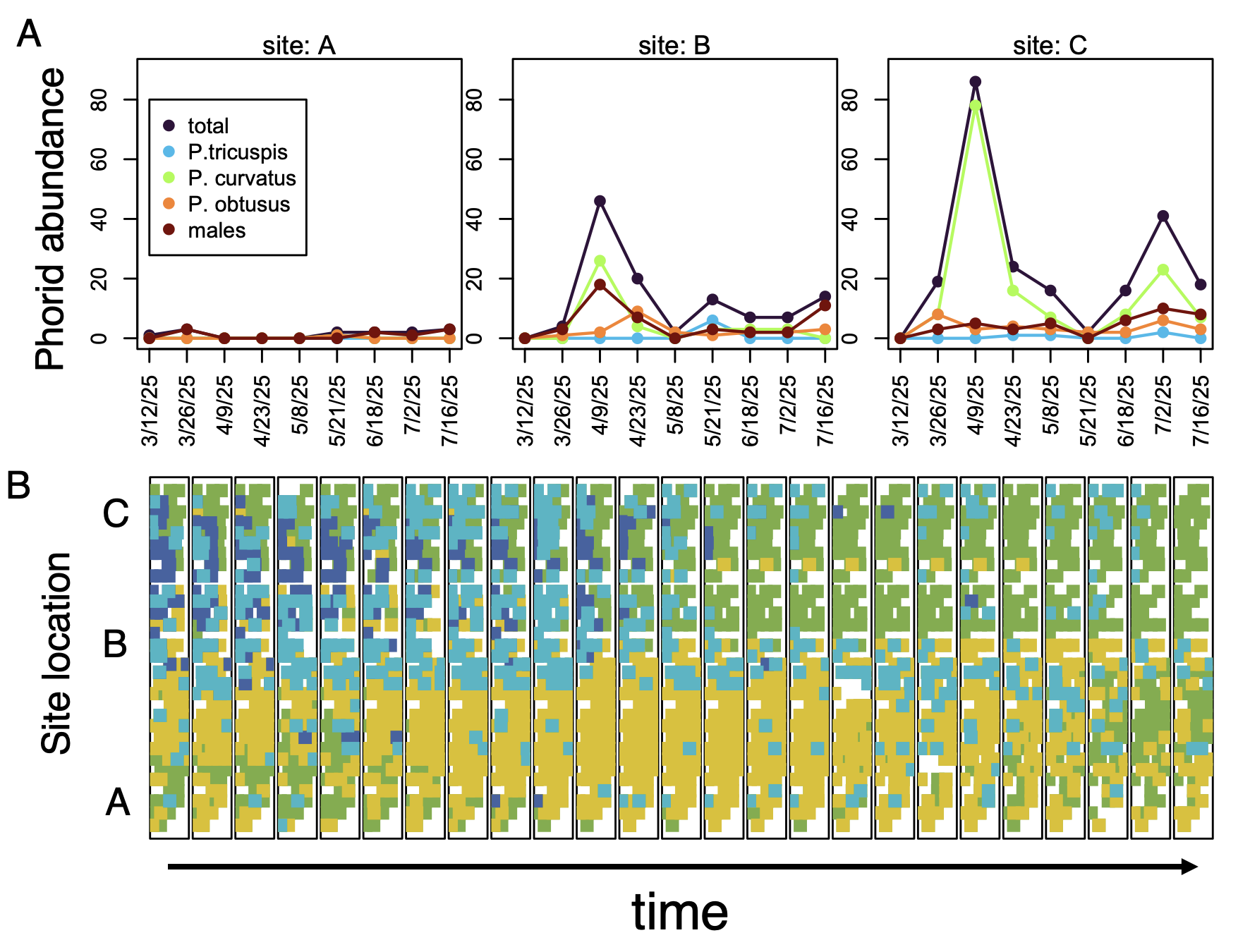


*Figure S2. A) Phorid abundance in three parts of the plot that correspond to* Solenopsis *dominance (red),* Wasmannia *dominance (green) and a contested territory between these two species. B) Time series of the plot for 25 monthly surveys showing the dominant ant species present (yellow =* Solenopsis*, Green =* Wasmannia*, teal =* Monomorium*, navy =* Technomyrmex*).*

**3. Modeling competitive dynamics of the ant community**

*i. Model description*

The cellular automata used in the study to simulate competitive dynamics of the ant community here was first described in detail in Vandermeer and Yitbarek (2012), and further studied in Vandermeer (2013) and Yitbarek and Vandermeer (2017). Additional details of the model, and its relationship to the classical Lotka-Volterra competition model can be found in the supporting information of Vandermeer and Yitbarek (2012).

The model is a spatially explicit cellular automata of competition that considers the presence and absence of species inside of patches on a lattice. Species diffuse locally in their Moore neighborhood (8 nearest patches) and when species neighbor each other in the Moore neighborhood, the occupancy of a focal patch is determined by the matrix of competitive effects between species. This model places emphasis on the competitive effects and responses of species within a community. The effect of species *i* on species *j*, or $\alpha_{ji}$, can be thought of as the probability that *i* will exclude *j*, while the response of *i* to *j*, or $\alpha_{ij}$, can be thought of as the probability that *i* will be excluded due to *j*. This can scale up to the row and column sums of the matrix **A** (Table S1) to understand the community-wide effects and responses of species in the community and the general structure of this has been explored previously (see Vandermeer and Yitbarek 2012 and Vandermeer 2013).

We formalize the effect competition of a species *i* at point (*m,n*) as,

$E_{i}\left( m,n \right)= \sum_{j=1}^{S} N_{i}^{'}\left( m,n \right)N_{j}^{'}\left( m,n \right)\alpha_{ji}$.

S is the total number of species in the system (here that is 5), $\alpha_{ji}$ is the competitive effect of species *i* on species *j* and $N_{i}^{'}$ represents the sum of occurrences of species *i,* in the Moore neighborhood of the focal patch (*m,n*) and can be written as

$N_{i}^{'}(m,n)= \sum_{x=m-1}^{m+1} \sum_{y=n-1}^{n+1} N_{i}\left( x,y \right)$,

Where *N_i_*(*x,y*) is the presence or absence of species *i*, at point *x,y*. We can then consider the spatially dependent response competition of species *i* with

$R_{i}\left( m,n \right)= \sum_{j=1}^{S} N_{j}^{'}\left( m,n \right)\alpha_{ij}$.

To determine the outcome of competition, and thus the occupant of the focal patch, we take the ratio of effect and response competition

$C_{i}\left( m,n \right)=\frac{E_{i}\left( m,n \right)}{R_{i}(m,n)}$,

and the species that has the highest value of C will then become the sole occupant of the patch *m,n.*

*ii. Model initialization and parametrization*

To initialize the model, we randomly seed a 100x50 cell lattice (reflecting the approximately rectangular shape of the study plot) with periodic boundaries where each species in the community has a 5% chance of being placed in every cell. While the determination of competitive outcomes as described above is deterministic, stochasticity enters into the model in its initialization as well as in the immigration dynamics of species. For each iteration in the model, there is a fixed probability that species *i* will colonize a random patch on the lattice. The immigration coefficients are informed from our empirical data and are calculated as the probability of a new tree being occupied by a species at survey month *t + 1* where it had been absent at month *t*. We estimate these values between surveys in our data, and although they are variable in time we use an average across time (Fig S3). The average values of immigration used in the model for the species are 0.2 for *Wasmannia*, 0.1 for *Solenopsis*, 0.05 for *Monomorium*, 0.035 for *Technomyrmex*, and 0.015 for the peripheral ant community.


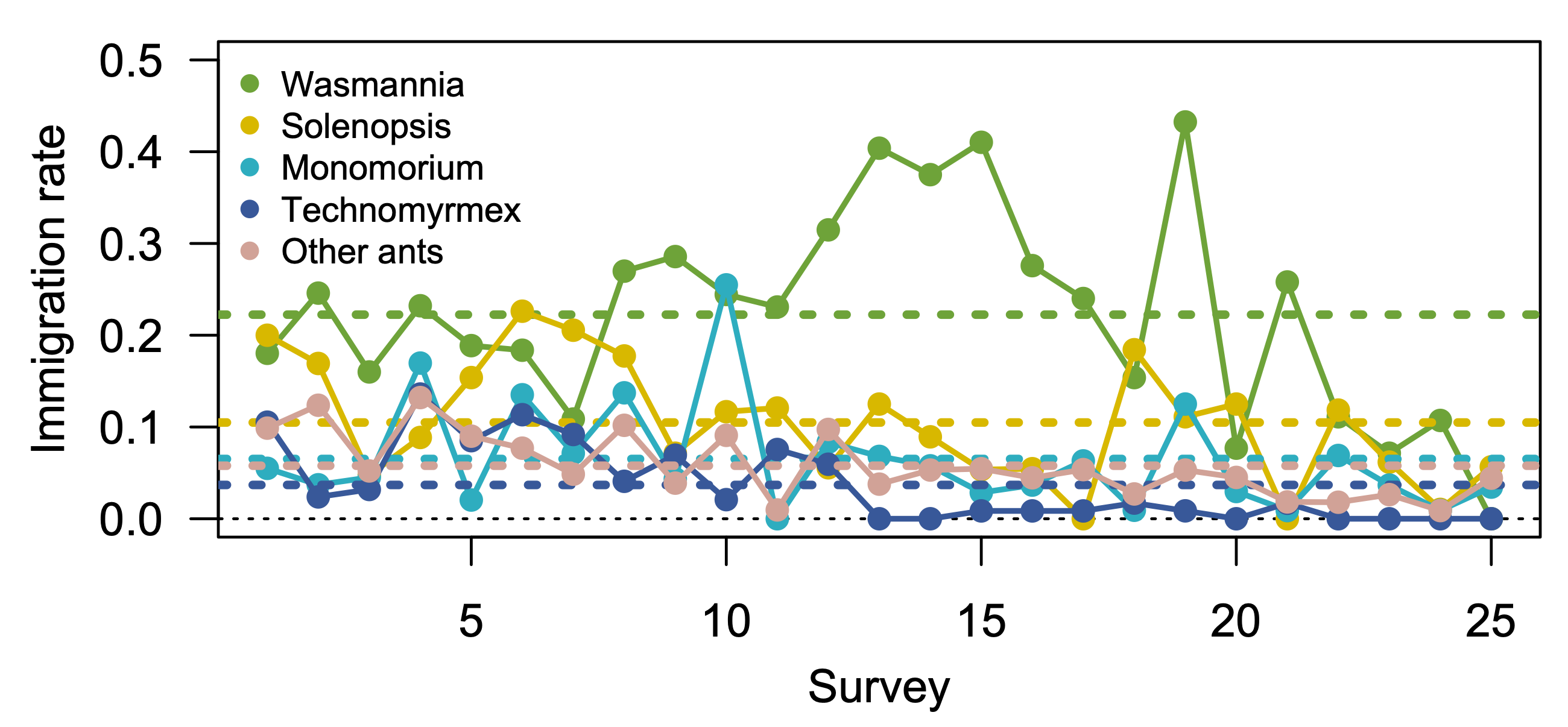


*Figure S3. Immigrations as calculated from the empirical data. The solid lines show the variation through time and the dashed lines show the average value which was used for the simulations.*

As mentioned in the main body of the manuscript, the HOI from the phorid parasitoids is known to exhibit complex oscillations through time, likely emerging from a combination of endogenous and exogenous factors on the phorid’s population dynamics. To understand the general effects of characteristics in the oscillations of phorids, and their implications for the coexistence of the community, we force the HOI with a sin function with different parameters. It takes the form HOI*(t) = A*Sin(f*t +v_offset_)+h_offset_*, where *A* stipulates the amplitude and *f* the frequency of the oscillations of HOI, and *h_offset_* and *v_offset_* are offsets for the *t* and HOI axis respectively (Fig S4). We set the *v_offset_* at the value of HOI which is at the center of the interregnum between the *Solenopsis* and *Wasmannia* regimes (middle section of Fig 5 in main body). The *Solenopsis* regime collapses at approximately HOI = 1.51 and the *Wasmannia* regime emerges approximately at HOI = 1.73, and the center point between these two regimes is 1.62 which is the value of the *v_offset_* used in all simulations. We set *h_offset_* = -1.2 to ensure that when simulations initialize the HOI starts at approximately the center of the interregnum value (~*v_offset_*) and subsequently increases. This ensures that when the HOI is at the peak of the oscillation, the *Wasmannia* regime will take over in the long run, and when the HOI is at its minimum in the oscillation that *Solenopsis* will take over in the long run. Below we show results from a range of frequencies and amplitudes of the HOI to understand how it shapes coexistence and community assembly of the system. Results of the model emerge from averaging 100 replicates of each parameter combination with random initial conditions, and each simulation is run for 500 timesteps.


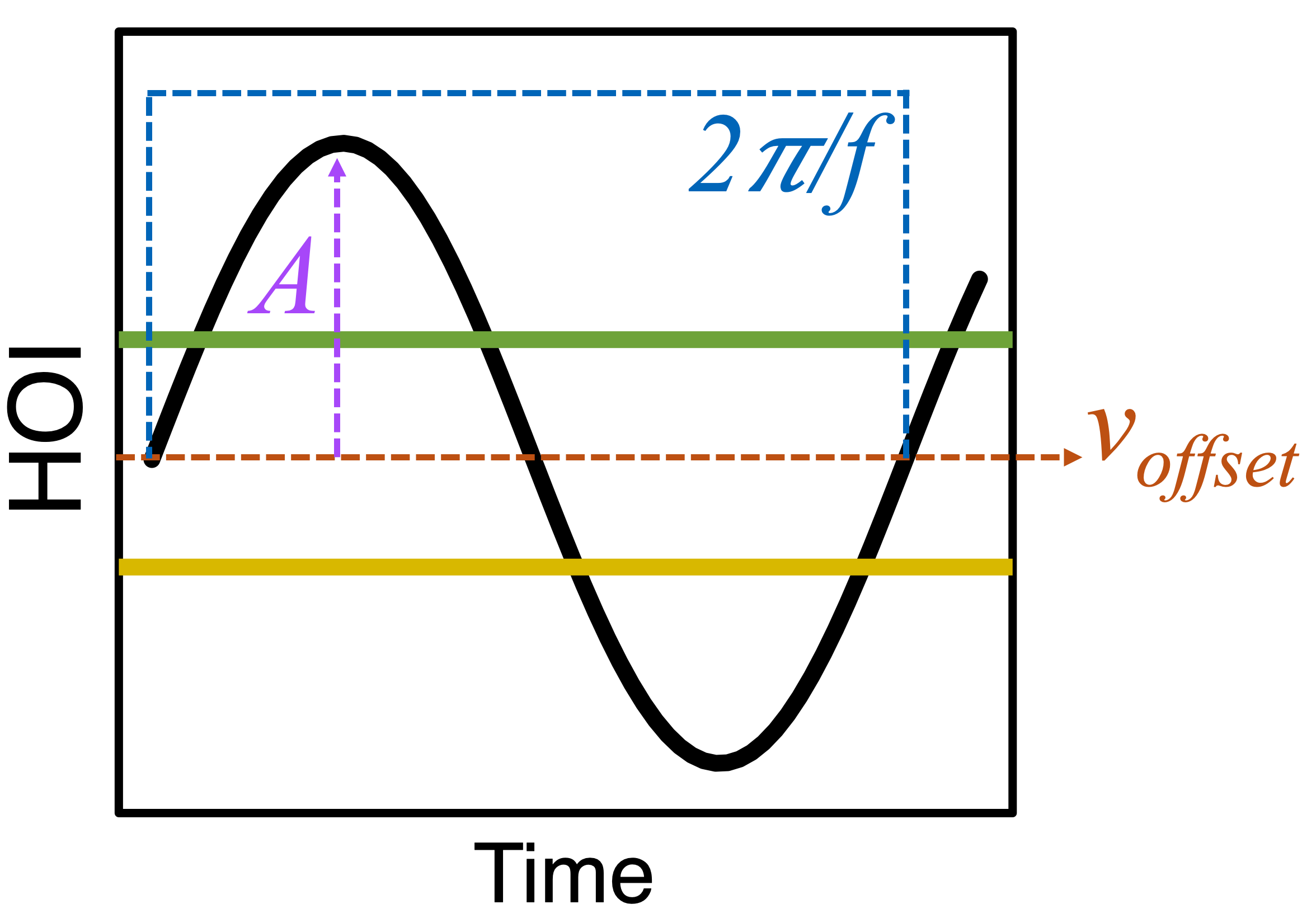


*Figure S4. Illustrates the parameters of the forced HOI oscillations in the model. Solid green line shows the approximate value of HOI for the onset of the* Wasmannia *regime, while solid yellow line shows the approximate value of HOI for the onset of the* Solenopsis *regime. V_offset_ shows the center point of the HOI function which falls at the center of the interregnum (space between* Wasmannia *and* Solenopsis *regimes). f shows the frequency, and A the amplitude of the HOI oscillations.*

*iii. Model dynamics*

In the main body of the manuscript, we show the qualitatively distinct scenarios that emerge from 3 different amplitudes and 3 different frequencies of the HOI (Fig 6). As previously noted, these simulations emerge from averaging the outcomes of 100 replicate simulations. Examples of time series from individual replicates are shown in Figure S5. While the averages across replicates give intuition about the general dynamics of the community in the face of a particular frequency and amplitude of HOI, the individual replicates show the variability in the dynamics. Note that although the oscillations appear very regular (Fig 6), there are irregular periods of time where one species may remain dominant in the community. Furthermore, the periodic appearance of *Technomyrmex* in the community appears much more clearly in the replicates than across the averages. While it is apparent that stochasticity in initial conditions and immigration plays a role in determining the realized dynamics of the model, the averages across multiple simulations show consistent patterns and differences between the different types of HOI oscillations.


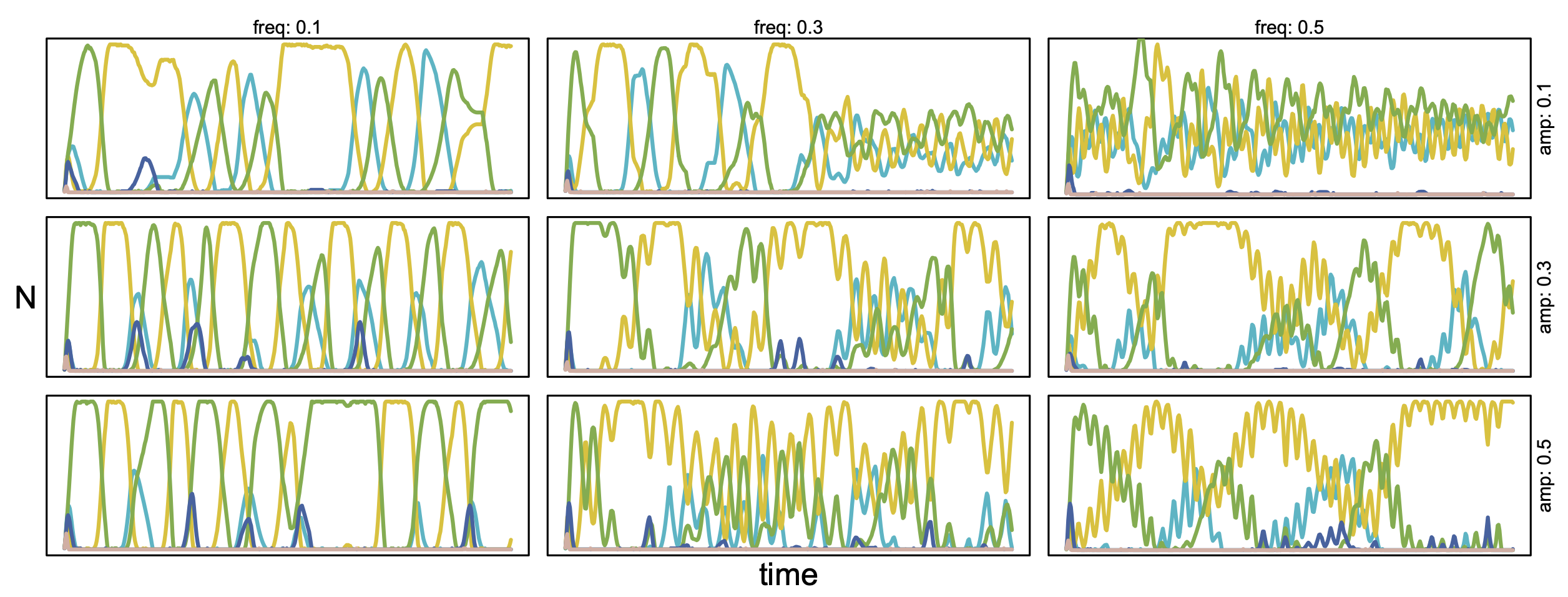


*Figure S5. Single replicates from the parameter combinations used in Figure 6 in the main body.*

The general effects of different frequency and amplitude of HOI across a wider range of values were also explored (Fig S6). We varied the HOI frequency from 0.1 to 0.9 by 0.1 and amplitude from 0.1 to 0.5 by 0.1. In the main text we show the different scenarios of frequency = 0.1,0.3,0.5, and amplitude = 0.1, 0.3,0.5. These combinations of parameters give a relatively complete picture of the dynamics of the community under different oscillatory dynamics of the HOI, as seen when the frequency surpasses 0.5, the qualitative results remain largely unchanged. This is also seen in the relative abundance of the species in the community (Fig S7).


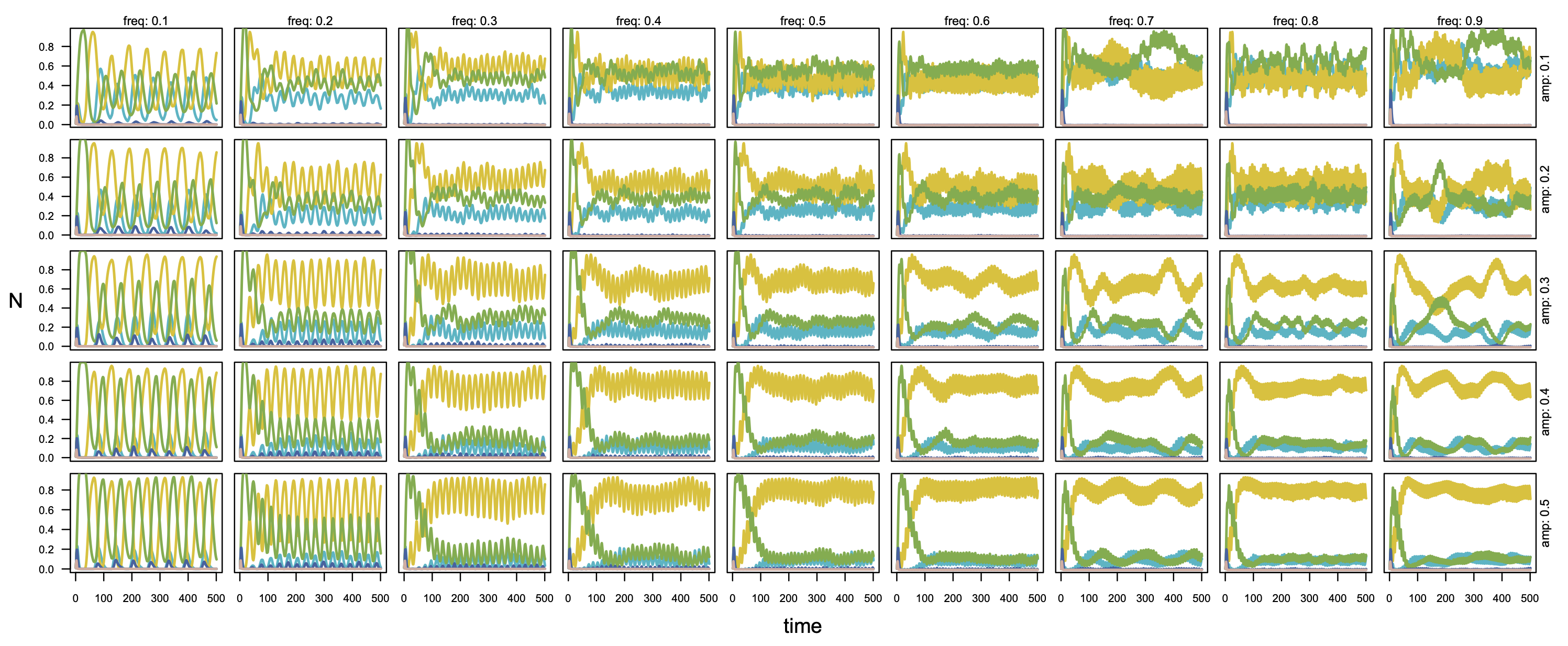


*Figure S6. Time series from the competition model with wider range of frequency and amplitude in HOI oscillation. Columns show different values of frequency and rows amplitude.*


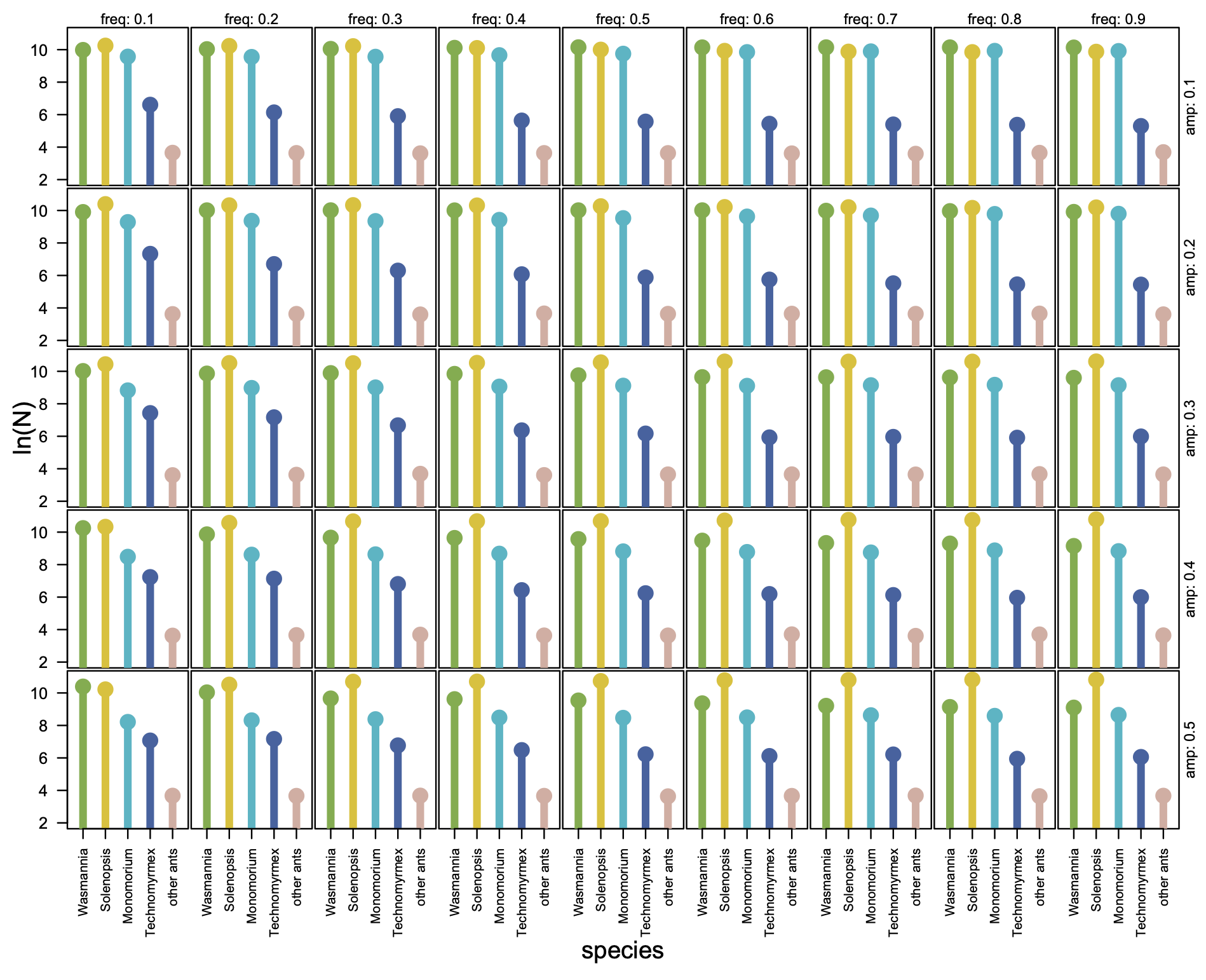


*Figure S7. Rank abundance distributions emerging from the model. Different columns show various frequencies of HOI oscillations and different rows show various amplitudes.*

**Literature cited**

Vandermeer, John, and Senay Yitbarek. "Self-organized spatial pattern determines biodiversity in spatial competition." *Journal of Theoretical Biology* 300 (2012): 48-56.

Vandermeer, John. "Forcing by rare species and intransitive loops creates distinct bouts of extinction events conditioned by spatial pattern in competition communities." *Theoretical ecology* 6, no. 4 (2013): 395-404.

Yitbarek, Senay, and John H. Vandermeer. "Reduction of species coexistence through mixing in a spatial competition model." *Theoretical Ecology* 10, no. 4 (2017): 443-450.
